# Mesh-sacropexy in women with apical pelvic organ prolapse – results of a single-centre prospective cohort study

**DOI:** 10.1186/s12905-026-04385-3

**Published:** 2026-03-12

**Authors:** Carolin Schröder, Jakob F. Pantenburg, Laura Tascón Padrón, Karla Feodorovici, Lucia A. Otten, Eva K. Egger, Sevinj Wittershagen, Alexander Mustea, Dominique Koensgen

**Affiliations:** 1 Department of Gynaecology and Gynaecological Oncology, University Hospital Bonn, Venusberg-Campus 1, Bonn, 53127 Germany; 2 Department of Gynaecology and Obstetrics, Havelland Kliniken Nauen and Rathenow, Ketziner Straße 21, 14641 Nauen and Forststraße 45, Rathenow, 14712 Germany

**Keywords:** Sacropexy, Mesh, Pelvic organ prolapse, Robotic-assisted, POP-surgery, Quality of life

## Abstract

**Background:**

Pelvic organ prolapse (POP) affects nearly half of women, highly influencing quality of life. When conservative treatment fails, surgical repair is indicated, with sacropexy representing the current gold standard for apical POP (aPOP) repair.

**Methods:**

Prospective single-centre cohort study including patients (pts) with aPOP and indication for surgery using polyvinylidene fluoride (PVDF) mesh (DynaMesh^®^) for sacrocervicopexy, sacrocolpopexy, or sacrohysteropexy at the University Hospital Bonn. Clinical data, peri- and postoperative outcomes, and follow-ups at 8 weeks (FU2), 6 months (FU3), and 12 months (FU4) postoperatively were analysed. The primary endpoint was the recurrence at FU4, defined as aPOP-Q ≥ grade II. Secondary endpoints included quality of life using the German Pelvic Floor Questionnaire (GPFQ), International Consultation on Incontinence Questionnaire – Urinary Incontinence Short Form (ICIQ-UI-SF), and a study-specific questionnaire.

**Results:**

A total of 90 women (median age 60 years [range 34–83], median body mass index 24.5 kg/m² [range 19.5–40.3]) underwent POP surgery between 2022 and 2024. Most procedures were robotic-assisted sacrocervicopexies (77.8%). Perioperative complications occurred in 4.4% (intraoperative) and 6.7% (postoperative) of cases. Median hospital stay was 5 days (range 3–9). Until FU4, no apical recurrences were observed; cumulatively, de-novo mesh erosion occurred in 4.4% of pts, with 2.2% requiring surgical revision. De-novo urinary incontinence was rare (5.5%). Subjective outcomes included scores of GPFQ and ICIQ-UI-SF, which improved significantly at all follow-up visits compared to preoperatively. 93% of pts were satisfied with the operation, 94% would choose it again, and 95% of pts would recommend this operation to a friend.

**Conclusions:**

Mesh-assisted sacropexy was associated with high apical success rates and favourable patient-reported outcomes at 12 months. Complication and reoperation rates were low within the observed follow-up period. However, longer-term data are required to fully evaluate durability and safety.

**Trial registration:**

Registration number: DRKS00038856. Registration date: 08th January 2026, registered retrospectively

## Introduction

Pelvic organ prolapse (POP) is a multifactorial disease affecting up to 40–60% of parous women [1]. Abdominal sacropexy has long been regarded as an established procedure for POP repair with reported success rates of around 91% [2]. Surgical treatment of POP follows a reconstructive and defect- as well as symptom-oriented approach aiming to restore anatomical support, pelvic floor function and to improve prolapse-related symptoms, including urinary or occult urinary incontinence.

Various surgical approaches have been described for the correction of POP, including abdominal, vaginal, combined and laparoscopic procedures. Based on earlier evidence, abdominal sacropexy was considered the gold standard for apical or combined defects [3]. As conventional abdominal sacropexy is associated with relevant morbidity and prolonged recovery, laparoscopic sacropexy has emerged as a minimally invasive procedure with lower morbidity, faster reconvalescence and comparable or possibly improved long-term success rates [4]. The 2016 and 2023 Cochrane reviews confirmed the superiority of sacropexy compared to vaginal approaches regarding clinical and subjective outcomes [5, 6]. Many studies indicate that the use of synthetic materials reduces the probability of prolapse recurrence, but at the same time increases the complication rate due to the use of synthetic materials [7]. While the use of synthetic mesh in urogynecology remains the subject of ongoing discussion, especially following FDA warnings on vaginal mesh procedures reporting erosion, pain and dyspareunia [8, 9], these concerns have also affected the perception of mesh-based abdominal procedures. Recently, the introduction of polyvinylidene fluoride (PVDF) meshes has attracted attention due to improved textile characteristics, enhanced biocompatibility, and a lower inflammatory response compared with conventional polypropylene meshes [10]. However, robust evidence regarding long-term safety, clinical outcomes and patient-reported outcomes after PVDF-based sacropexy is still limited. To date, most studies assessing sacropexy are retrospective with small patient cohorts and lack systematic long-term follow-up. This prospective study aimed to analyse objective anatomical outcomes and subjective patient-reported outcomes, including quality of life and sexual function, following mesh-based apical POP (aPOP) repair using PVDF.

## Materials and methods

### Study design

Prospective, single-centre cohort study, including 90 pts who underwent surgery for aPOP at the Department of Gynaecology and Gynaecologic Oncology, University Hospital Bonn, between 2022 and 2024. In all cases, mesh implants made of PVDF material (DynaMesh^®^, FEG Textiltechnik mbH, Aachen, Germany) were used for sacrocervicopexy, sacrocolpopexy, or sacrohysteropexy. POP was staged according to the Pelvic Organ Prolapse Quantification (POP-Q) system [11]. Pts aged 18 years or older with aPOP stage I or higher who gave their informed consent were included. Conservative treatment measures were recommended to all pts. Pts with grade I prolapse were included only if they were highly symptomatic and had failed conservative treatment. The primary endpoint was the objective outcome at 12 months; recurrence was defined as aPOP-Q ≥ grade II. Secondary endpoints included quality of life (GPFQ, ICIQ-UI-SF), complication rates, and patient satisfaction.

### Clinical data

All pts underwent preoperative urodynamic testing to assess the need for concomitant incontinence procedure. Clinical data included age, body mass index (BMI, kg/m²), ASA classification, parity, history of prolapse or incontinence surgery, prior hysterectomy, intra- and postoperative complications, and length of hospital stay.

### Surgical technique of robotic sacrocervicopexy

**Fig. 1:**
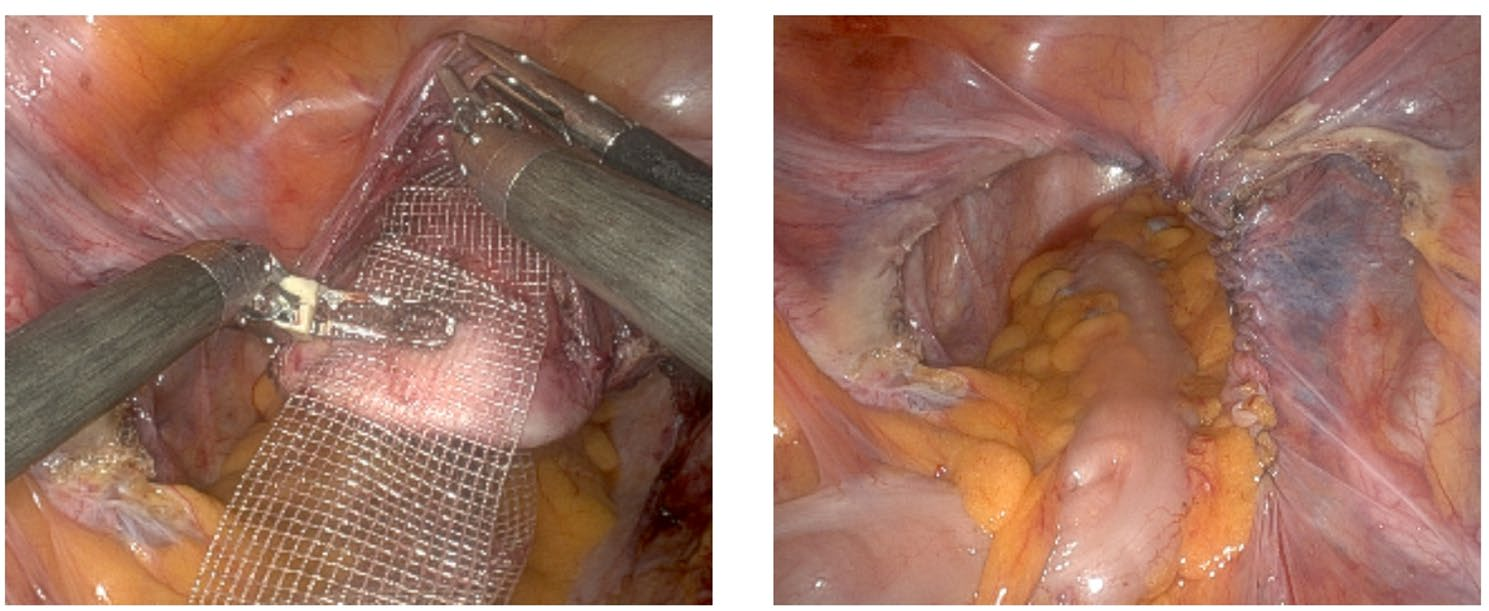
Intraoperative imaging of the situs - left: placement of PRS-MESH (Y-shaped), right: after re-peritonealisation

Under general anaesthesia, the patient is positioned in lithotomy with Trendelenburg tilt, and both a bladder catheter and HOHL uterine manipulator are inserted. Robotic-trocars are inserted at the umbilical (camera), 8 cm left and right of the umbilical, and an assist-trocar is positioned 8 cm lateral to the right trocar. Supracervical hysterectomy is conducted, including coagulation and division of the round ligaments and uterine arteries, separation of the bladder, and morcellation of the uterine corpus within an endobag. Additional salpingectomy or adnexectomy is performed according to menopausal status. For sacrocolpopexy, the peritoneum over the promontory on L1/S1 and along the sacrouterine ligaments is opened, the bladder and rectum are dissected off the vaginal walls, and a mesh is positioned. The PRS-mesh is fixed to the anterior and posterior vaginal wall with 5–10 PDS sutures, at the cervical stump using four Ethibond sutures, and to the longitudinal ligament using two Ethibond sutures. PRS Dynamesh is a Y-shaped mesh that is used when the pt has a multi-compartment defect in the anterior and posterior compartments. Without prolapse of the posterior compartment, PRR-/PR Dynamesh is used and fixed to the anterior wall. Concomitant, anterior or posterior colporrhaphy can be performed as needed by approximating the paravaginal tissue either transversely and/or lengthwise using single-stitch sutures. Resorbable materials such as PDS 2 − 0 or Vicryl 2 − 0 are commonly used. Re-peritonealisation is conducted using V-Loc suture (Fig. 1). Additionally, incontinence procedures such as Burch colposuspension (BC) and tension-free vaginal tape (TVT) are performed when indicated.

### Follow-up

Complications were classified according to the Clavien–Dindo system [12]. Follow-up evaluations were conducted at discharge (FU1), 8 weeks (FU2), 6 months (FU3), and 12 months (FU4) postoperatively.

Postoperatively, pts received a permanent urinary catheter, which was removed after three days (after two days in Burch procedures and one day in TVT). Vaginal tamponade with estrogen ointment was placed for 24 h postoperatively. All pts received standard perioperative antibiotic prophylaxis according to institutional protocol (cefuroxime/clindamycin + metronidazole). Subjective outcomes and quality of life were assessed using the German Pelvic Floor Questionnaire (GPFQ) and the International Consultation on Incontinence Questionnaire – Urinary Incontinence Short Form (ICIQ-UI-SF). The GPFQ comprises four domains: bladder function (15 items), bowel function (12 items), prolapse symptoms (5 items), and sexual function (3–10 items, depending on sexual activity). Each domain is scored up to 10 points (possible total score 0–40), with higher scores indicating poorer pelvic floor function [13]. The ICIQ-UI-SF assesses frequency of incontinence, amount of leakage, and overall impact, and includes a self-diagnostic item, with total scores ranging from 0 to 21 (higher scores indicating greater severity) [14]. Additionally, a study-specific questionnaire (SSQ) captured patient-reported outcomes such as perceived surgical success, satisfaction, willingness to undergo the same procedure again, recommendation to others, and postoperative changes and sexual function. Percentage data from the SSQ is based on the number of fully completed questionnaires.

### Statistical analysis

Statistical analysis was performed using SPSS 29.0 (IBM Corp., Armonk, NY, USA). Descriptive statistics were used to summarise patient characteristics, surgical data, and peri- and postoperative outcomes. Changes in POP-Q stage, GPFQ, and ICIQ-UI-SF scores were analysed using the Friedman test with post hoc Wilcoxon signed-rank tests. Categorical variables were compared using the Chi-square test or Fisher’s exact test, as appropriate. Continuous variables were analysed using the Mann–Whitney U test. A two-sided p-value < 0.05 was considered statistically significant. The study was designed as an exploratory prospective cohort study, without any power calculation. Ethical approval was obtained from the local ethics committee at the University of Bonn (reference number 289/21). This study was registered in the German Clinical Trials Register (DRKS) under the number DRKS00038856 on 8th January 2026. The study was registered retrospectively due to administrative reasons. However, the study protocol and endpoints were prospectively defined and approved by the local ethics committee.

## Results

### Patient characteristics

A total of 90 women with symptomatic aPOP were included between 2022 and 2024 at the Department of Gynaecology and Gynaecologic Oncology at the University Hospital Bonn. The median age at the time of surgery was 60 years (range 34–83). The median body mass index (BMI) was 24.5 kg/m² (range 19.5–40.3). Most women (88/90; 97.8%) had at least one prior vaginal delivery, with a median parity of 2 (range, 0–6). A previous gynecologic surgery had been performed in 47 pts (52.2%), and a hysterectomy (any approach) in 14 pts (15.6%). Previous anterior colporrhaphy had been performed in 6 pts (6.7%), posterior colporrhaphy in 3 (3.3%), and TVT in 1 pt (1.1%). Baseline incontinence assessment revealed mixed urinary incontinence (MUI) in 35 pts (38.9%), stress urinary incontinence (SUI) in 25 (27.8%), urge urinary incontinence (UUI) in 10 (11.1%), and no preoperative incontinence symptoms in 20 pts (22.2%).

All pts presented with symptomatic aPOP. The leading symptom was vaginal bulge and foreign body sensation, reported by 79 pts (87.8%). Additional symptoms included voiding dysfunction in 12 pts (13.3%), recurrent urinary tract infections in 8 (8.9%), and chronic constipation in 2 (2.2%). In the apical (middle) compartment, 7 pts (7.8%) had stage I prolapse, 51 pts (56.7%) had stage II prolapse, 26 pts (28.9%) had stage III prolapse, and 6 pts (6.7%) had stage IV prolapse. Multicompartment involvement was frequent: 28 pts (31.1%) had clinically relevant POP (stage ≥ II) in all three compartments, 50 (55.6%) in two compartments, and 12 (13.3%) only in the apical compartment. Patient’s characteristics are summarised in Table 1.

**Table 1:** Patient characteristics

Age years median (range)	60 (34-83)
BMI kg/m^2^ median (range)	24.5 (19-40.3)
Vaginal delivery (≥ 1) n (%)	88 (97.8)
Number of vaginal deliveries median (range)	2 (0-6)
Sexually active n (%)	
FU2	32 (35.6)
FU3	31 (34.4)
FU4	42 (46.7)
Previous urogynecological surgery n (%)	47 (52.2)
Anterior colporrhaphy	6 (6.7)
Posterior colporrhaphy	3 (3.3)
TVT	1 (1.1)
Previous hysterectomy n (%)	14 (15.6)
Type of incontinence n (%)	
Mixed incontinence	35 (38.9)
Stress incontinence	25 (27.8)
Urge incontinence	10 (11.1)
None	20 (22.2)
Leading symptoms n (%)	
Vaginale bulge	79 (87.8)
Voiding dysfunction	12 (13.3)
Recurrent urinary tract infections	8 (8.9)
Chronic obstipation	2 (2.2)
aPOP grade n (%)	
Grade I	7 (7.8)
Grade II	51 (56.7)
Grade III	26 (28.9)
Grade IV	6 (6.7)
Multicompartment distribution n (%)	
One / apical compartment	12 (13.3)
Two compartments	50 (55.6)
Three compartments	28 (31.1)

### Surgical data

The surgery was performed robotically in 87 pts (96.7%), by laparotomy in 3 pts (3.3%). Of these, 2 pts (2.2%) had planned laparotomy, and in 1 patient (1.1%) conversion to laparotomy was necessary due to adhesions. The type of apical fixation included sacrocervicopexy in 70 pts (77.8%), sacrocolpopexy in 13 (14.4%), sacrohysteropexy in 6 (6.7%), and concomitant anterior rectopexy in 1 patient (1.1%). The most frequently used mesh type was DynaMesh^®^-PRS soft (45/90; 50.0%), followed by DynaMesh^®^-PRR soft (42/90; 46.7%) and DynaMesh^®^-PR soft (3/90; 3.3%). Anterior colporrhaphy was performed in 24 pts (26.7%), posterior colporrhaphy in 11 pts (12.2%), and Burch colposuspension in 34 pts (37.8%). Surgical data are summarised in Table 2.

**Table 2:** Surgical data

Surgical assess n (%)	
Robotic-assisted	87 (96.7)
Open	3 (3.3)
Surgical procedure n (%)	
Sacrocervicopexy	70 (77.8)
Sacrocolpopexy	13 (14.4)
Sacrohysteropexy	6 (6.7)
DynaMesh n (%)	
PRS	45 (50)
PRR	42 (46.7)
Additional procedure n (%)	
Anterior	24 (26.7)
Posterior	11 (12.2)
Burch colposuspension	34 (37.8)
Hospital stay days median (range)	5 (3-9)

### Complications

Intra- and postoperative complications were rare according to the Clavien-Dindo classification (Table 3). All surgeries were completed successfully according to protocol. Reoperation was necessary in 1 patient (1.1%). The median postoperative hospital stay for the entire cohort was 5 days (range 3–9).

**Table 3:** Intra- and postoperative complications (according to the Clavien–Dindo classification)

Complication type	*n* (%)	Description	Clavien–Dindo grade^a^ (12)
Intraoperative complications	4 (4.4%)		
Iatrogenic intestinal lesion	2 (2.2%)	Minor bowel injury, treated intraoperatively, no conversion required	I
Cardiac complication	1 (1.1%)	Transient cardiac AV block during surgery, resolved spontaneously	I
Subcutaneous emphysema	1 (1.1%)	Mild CO₂ emphysema, resolved spontaneously	I
Postoperative complications (until 8 weeks postoperative)	6 (6.7%)		
Haematoma	3 (3.3%)	One case required surgical revision, others conservative treatment; during initial hospital stay	IIIb (*n* = 1), II (*n* = 2)
Fever of unknown origin	1 (1.1%)	Empiric antibiotic therapy, full recovery; during initial hospital stay	II
Obstipation / Ileus	1 (1.1%)	Pharmacological treatment, re-admission needed	II
Nephrolithiasis, hydronephrosis	1 (1.1%)	Pharmacological treatment, re-admission needed	II

^a)^ Clavien-Dindo classification - *grade I* no pharmacological or surgical intervention, *grade II* pharmacological treatment, *grade III* surgical, endoscopic, or radiological intervention (IIIa without, IIIb with general anaesthesia), *grade IV* life-threatening/intensive care management, *grade V* death

### Objective outcome

Follow-up examinations were performed at FU2 (median 2 months, range 1–6), FU3 (median 6 months, range 1–9) and FU4 (median 12 months, range 10–18). The lost-to-follow-up rate was 2% at FU2 and 11% at FU3 and FU4. *Recurrence rate*: POP-Q grades were significantly reduced in all three compartments at FU1, FU2, FU3 and FU4 compared with preoperatively (all *p* < 0.001, Table 4). No apical prolapse recurrence ≥ grade II was observed during FU. Of the 15 pts with anterior compartment prolapse ≥ grade I at FU4, 13 (87%) had an anterior prolapse preoperatively, while 2 (2.5%) represented de-novo anterior prolapse (1 pt after PRS-mesh, 1 pt after PRR-mesh). Of the 28 pts with posterior prolapse ≥ grade I at FU4, 23 (82%) were recurrences, and 5 (17.9%) were de-novo posterior prolapse (1 pt with PR and PRS each, 3 pts with PRR). Of those 28 pts, 27 (96.4%) received sacrocervicopexy, 1 pt sacrocolpopexy (3.6%). Of those 28 pts, PR-mesh was used in 2 pts, PRR and PRS in 13 pts each.

**Table 4:** Objective Outcome according to POP-Q ≥ grade II

Compartment	Preoperative (*n* = 90)	FU1 (*n* = 90)	FU2 (*n* = 88)	FU3 (*n* = 80)	FU4 (*n* = 80)
Anterior	76 (84.4)	1 (1.1)^a^	1 (1.1)^a^	1 (1.3)^a^	4 (5)^b^
Apical	83 (92)	0 (0)	0 (0)	0 (0)	0 (0)
Posterior	37 (41.1)	0 (0)	0 (0)	1 (1.3)^a^	2 (2.5)^c^

^a)^ 1 pt grade II (without colporrhaphy, PRS-mesh)

^b)^ 3 pts grade II (2 pts with anterior colporrhaphy, 1 pt without, all PRR-mesh), 1 pt grade IV (without colporrhaphy, PRS-mesh

^c)^ 2 pts grade II (1 pt with posterior colporrhaphy, 1 pt without, both PRR-mesh)

### Reoperation rate

At FU2, 2 pts (2.2%) had de-novo erosion, with no need for surgical revision (both after sacrocervicopexy, 1 PRR-mesh, 1 PRS-mesh). At FU3, 1 pt (1.1%) with additional de-novo mesh erosion underwent surgery. This pt had a previous sacrocolpopexy using PRS-mesh. At FU4, 2 pts (2.2%) had erosion; one of these pts already had erosion at FU2 and was treated for erosion and rectocele II° after FU4; this pt had a sacrocervicopexy with PRS-mesh before. The other pt with erosion at FU4 had de-novo erosion, was treated conservatively with local oestrogen, and had a previous sacrocolpopexy using PRS-mesh. Cumulatively until FU4, de-novo mesh erosion occurred in four pts (4.4%). Surgical revision for erosion was required in two pts (2.2%).

### Incontinence

At FU2, 2 pts (2.2%) reported de-novo SUI and 2 pts (2.2%) de-novo UUI. All pts were treated conservatively. At FU3, 2 pts (2.2%) complained of de-novo SUI and 2 pts (2.2%) of UUI, neither of which was present at FU2. In one of those pts, SUI was treated conservatively, but this pt received surgery for erosion. At FU3, 1 pt (1.1%) received TVT for MUI, which had been persistent since preoperative. At FU4, 1 pt (1.1%) had de-novo SUI and 1 pt (1.1%) had de-novo UUI, both treated conservatively. One pt (1.1%) received TVT for SUI, which had been persistent since preoperative. Until FU4, overall de-novo SUI occurred in 5 pts (5.5%) with no need for incontinence surgery. Until FU4, 2 pts (2.2%) received surgery for SUI that had been clinically apparent preoperatively.

At FU4, objective outcomes did not differ meaningfully between pts with preoperative stage I–II and stage III–IV apical prolapse, with no apical recurrences observed in either group. Subjective outcomes were largely comparable between groups; only the ICIQ-UI-SF score differed statistically, while overall satisfaction and willingness to undergo surgery again were similarly high in both cohorts (Table 5).

**Table 5:** Objective Outcome at FU4 stratified by apical prolapse stage (stage I-II vs. stage III-IV preoperative)

Objective Outcome	Preoperative stage I-II *n* = 58	Preoperative stage III-IV *n* = 32	*p*-value
Anterior recurrence ≥ stage II	3 (5.2%)	1 (3.1%)	*N.A.*
Apical recurrence ≥ stage II	0	0	*N.A.*
Posterior recurrence ≥ stage II	1 (1.7%)	1 (3.1%)	*N.A.*
Any compartment ≥ stage II	4 (6.9%)	2 (6.3%)	*N.A.*
Multicompartment ≥ stage II	0	1 (3.1%)	*N.A.*
Reoperation (any reason)	3 (5.2%)	1 (3.1%)	*N.A.*
Mesh erosion (de-novo)	2 (3.4%)	1 (3.1%)	*N.A.*
Subjective Outcome			
GPFQ total score, median, [IQR]	19.0 [15.0] (*n* = 39)	13.0 [11.5] (*n* = 22)	*0.122*
GPFQ prolapse domain, median, [IQR]	0 [2.0] (*n* = 47)	0 [0.0] (*n* = 25)	*0.115*
ICIQ-UI-SF score, median, [IQR]	4.0 [8.0] (*n* = 50)	2.0 [4.0] (*n* = 26)	***0.027***

*N.A.* not applicable, *IQR* interquartile range

Subjective outcome denominators vary due to incomplete questionnaire responses at FU4; medians are calculated based on available data. *P*-values for subjective scores were calculated using the Mann–Whitney U test

Descriptive comparison of outcomes after PRS- and PRR-mesh is summarised in Table 6. Due to small subgroup sizes, no formal statistical comparison was performed.

**Table 6:** Outcome PRS vs. PRR

DynaMesh	PRS, n (%)	PR/PRR, n (%)
	45 (50)	45 (50)
POP-Q ≥ grade II at FU2 (n = 88)		
Anterior	1 (2.2)	0 (0)
Apical	0 (0)	0 (0)
Posterior	0 (0)	0 (0)
POP-Q ≥ grade II at FU3 (n = 80)		
Anterior	1 (2.2)	0 (0)
Apical	0 (0)	0 (0)
Posterior	1 (2.2)	0 (0)
POP-Q ≥ grade II at FU4 (n = 80)		
Anterior	1 (2.2)	3 (6.7)
Apical	0 (0)	0 (0)
Posterior	0 (0)	2 (4.4)
De-novo prolapse at FU4 (n = 80)		
Anterior n = 2	1 (2.2)	1 (2.2)
Apical	0 (0)	0 (0)
Posterior n = 5	1 (2.2)	4 (8.9)
Mesh erosion at FU2 (n = 88)	1 (2.2)	1 (2.2)
Reoperation	0 (0)	0 (0)
Mesh erosion at FU3 (n = 80)	1 (2.2)	0 (0)
Reoperation	1 (2.2)	0 (0)
Mesh erosion at FU4 (n = 80)	2 (4.4)*	0 (0)
Reoperation	1 (2.2)	0 (0)
Clavien-Dindo ≥ grade II		
Intraoperative (n = 4, 4.4%)		
Mesh related	0 (0)	0 (0)
Postoperative (n = 6, 6.7%)		
Mesh related	0 (0)	0 (0)
Study specific questionnaire at FU4		
Patients’ satisfaction	32 (71.1)	35 (77.8)
Would choose operation again	33 (73.3)	35 (77.8)
Would recommend operation	33 (73.3)	36 (80)

*one pt with persistent mesh-erosion since FU2, one pt with de-novo

### Subjective outcome

**Fig. 2:**
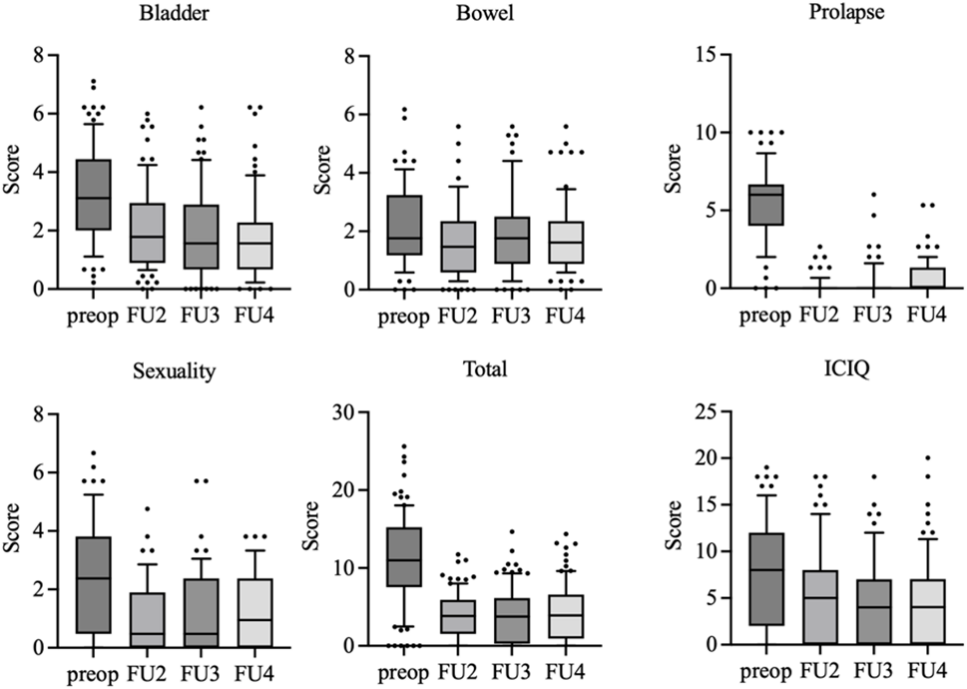
Results of GPFQ and ICIQ-UI-SF. Box plots of symptom scores of GPFQ (bladder, bowel, prolapse, sexuality, total) and ICIQ-UI-SF compared between preoperative, FU2, FU3 and FU4

Subjective outcomes included scores of GPFQ and ICIQ-UI-SF, which improved significantly at all FU visits compared with preoperatively (*p* < 0.05, Fig. 2).

### Study-specific questionnaire (Fig. 3)

At FU4, based on 72 available questionnaires, 67 pts (93.1%) were satisfied with the operation, 68 pts (94.4%) would choose it again, and 69 pts (96%) would recommend it to a friend. Among sexually active women at FU4 (*n* = 42, 46.7%), 95% reported satisfaction with the surgical outcome. 24 pts were sexually inactive at FU4; of those, 91% reported satisfaction with the surgical outcome. Postoperative satisfaction did not differ significantly between sexually active and inactive pts (Fisher’s exact test, *p* = 0.61). OAB symptoms were reported by up to 75% of pts preoperatively. These improved in half of the pts, but recurred even more frequently during the course of FU.

**Fig. 3:**
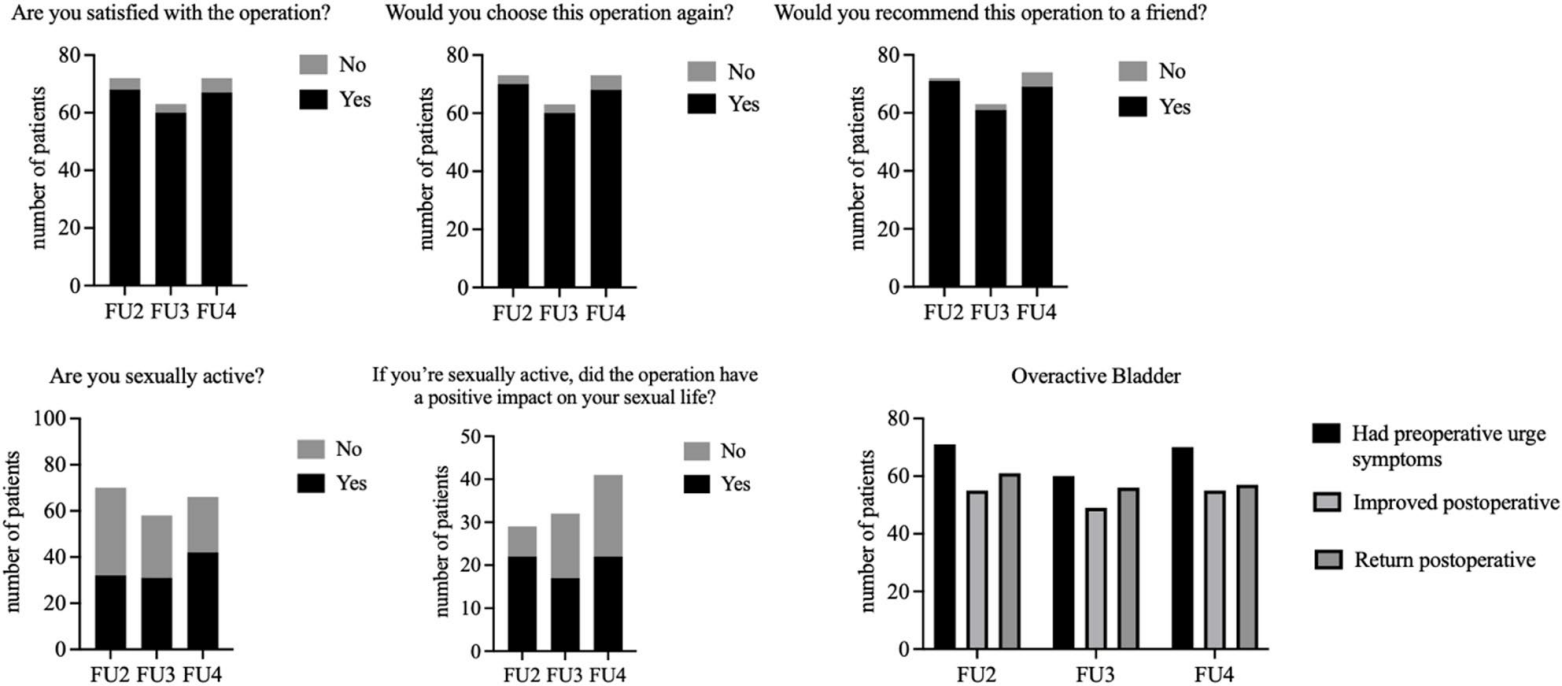
Results of SSQ. Bar chart from the results of the SSQ at FU2, FU3, and FU4

## Discussion

This prospective cohort study evaluated objective and subjective outcomes in pts with aPOP treated with mesh-based sacropexy using PVDF material. Our findings suggest that this approach provides favourable anatomical repair, high patient satisfaction, and significant improvement in quality of life, with low complication and anterior and posterior recurrence rates over a 12-month follow-up period.

The anatomical success rate in our cohort was very high, with no apical recurrences, low recurrences in the anterior and posterior compartments, and a reoperation rate below 5%, aligning with published long-term success rates of 85–95% for abdominal sacropexy procedures [15, 16]. POP-Q grades significantly improved across all compartments, confirming the good mid-term reconstructive potential of mesh-based fixation. Subjectively, pts reported a marked improvement in bladder, bowel, and prolapse symptoms, as assessed by the GPFQ and ICIQ-UI-SF, consistent with the significant functional restoration expected after sacropexy.

Notably, more than 90% of pts reported high satisfaction and a willingness to recommend or repeat the procedure, indicating favourable patient-reported outcomes at 12 months. Our results are in line with previous studies showing superior anatomical durability and lower recurrence rates after abdominal compared to vaginal approaches [17, 18]. Several randomised and cohort trials have confirmed that abdominal or robotic-assisted sacropexy achieves better apical support and fewer reoperations than native-tissue vaginal repairs, albeit at the cost of longer operative time [16, 17]. 

The low complication rate observed in our study (4.4% intraoperative, 6.7% postoperative) compares favourably with international registry data, such as the VIGI-MESH registry, which reported serious adverse events in up to 9% of cases [7]. The use of PVDF mesh may contribute to this safety profile, as it is characterised by higher biostability, a reduced inflammatory response, and lower shrinkage than polypropylene meshes [10]. Cumulatively until FU4, de-novo mesh erosion occurred in 4.4% of pts, with 2.2% requiring surgical revision. These rates are comparable to those reported in previous abdominal sacropexy series [19]. 

Our findings suggest a trend towards improved postoperative sexual function among sexually active women. However, sexual function was not assessed using a dedicated validated instrument specifically designed to evaluate sexual function. In addition, only 46.7% of pts were sexually active at FU4, which limits the generalisability of these findings. While the regression model did not identify sexual activity as an independent predictor of satisfaction, the observed trend should be interpreted cautiously. Potential anatomical factors influencing postoperative sexual function – such as preserved vaginal axis and length after laparoscopic sacropexy found in comparable studies- were not specifically evaluated in this study [18]. Previous studies have shown that abdominal sacropexy is associated with lower rates of dyspareunia and better sexual satisfaction, in comparison to vaginal mesh or native-tissue repairs [17, 18]. The higher proportion of sexually active pts at FU4 (46.7%) compared to FU3 (34.4%) may reflect postoperative recovery over time as well as variability in FU participation.

In a nationwide cohort study (FINPOP), POP surgery with a concomitant SUI procedure resolves or improves SUI symptoms in approximately half of women with preoperative SUI; de-novo SUI develops in about 20% of previously continent women, but bothersome symptoms and the need for intervention are rare. High baseline SUI severity increases the risk of persistent SUI, whereas advanced apical POP is associated with a lower risk of persistent SUI after surgery [20]. The incidence of de-novo SUI in our cohort (5.5%) was lower than that reported in the FINPOP study, but comparable to or lower than in other series [15, 21, 22]. However, our study was not powered to evaluate the de-novo SUI rate.

Interestingly, minor discrepancies between clinical interviews and questionnaire-based reports highlight the importance of integrating both objective and patient-reported outcome measures when assessing surgical efficacy. This discrepancy emphasises the importance of incorporating patient-reported outcomes, as studies have shown that questionnaires often indicate success rates up to 25% lower than those reported by surgeons, with Lebret et al. reporting a similar 18% gap [23, 24]. This dual assessment represents a strength of the present study, as it captures both the anatomical (objective) and psychosocial (subjective) dimensions of surgical success.

The strengths of this trial include its prospective design, standardised follow-up, and comprehensive evaluation using validated questionnaires (GPFQ, ICIQ-UI-SF, and SSQ). Furthermore, the exclusive use of PVDF mesh enables a homogeneous material-based assessment, minimising confounding from mesh heterogeneity. However, this study has several limitations. The cohort comprised pts with varying degrees of prolapse severity, different surgical histories including prior hysterectomy, and both isolated apical and multicompartment defects. In addition, different operative techniques were applied (sacrocervicopexy, sacrocolpopexy, and uterine-preserving procedures), reflecting routine clinical practice but potentially affecting subgroup comparability. The overall sample size was moderate, and it was not powered to detect low apical recurrence rates, limiting the statistical strength of subgroup analyses. Although follow-up compliance was high (89% at 12 months), longer-term data are required to assess durability beyond the first postoperative year. Moreover, a relevant proportion of pts received concomitant procedures, such as colporrhaphy and BC, which may have contributed to the observed anatomical and functional outcomes. Additionally, although subjective outcomes were comprehensively evaluated, the absence of a control group (e.g., native tissue repair or other mesh materials) limits the ability to draw comparative conclusions.

## Conclusion

Overall, mesh-assisted sacropexy using PVDF appears to be a safe and effective option for apical POP repair, demonstrating favourable anatomical and patient-reported outcomes at 12 months. Future multicentre randomised trials with longer follow-up are warranted to validate these findings and further elucidate the role of PVDF mesh compared with other synthetic or biological materials.

## Data Availability

Dataset and materials used in this study are available from the corresponding author upon reasonable request.

## Abbreviations

aPOP: Apical pelvic organ prolapse
BC: Burch colposuspension
BMI: Body mass index
FINPOP: Finnish Pelvic Organ Prolapse study
FU1: Follow–up at discharge
FU2: Follow–up at 8 weeks
FU3: Follow–up at 6 months
FU4: Follow–up at 12 months
GPFQ: German Pelvic Floor Questionnaire
ICIQ: UI–SF–International Consultation on Incontinence Questionnaire–Urinary Incontinence Short Form
MUI: Mixed urinary incontinence
OAB: Overactive bladder
PDS: Polydioxanone suture
POP: Pelvic organ prolapse
POP: Q–Pelvic Organ Prolapse Quantification
PR: Anterior Dynamesh
PRR: Anterior Dynamesh (PRR type)
PRS: Posterior/anterior Y–shaped Dynamesh
PT: Patient
PTS: Patients
PVDF: Polyvinylidene fluoride
SD: Standard deviation
SPSS: Statistical Package for the Social Sciences
SSQ: Study–specific questionnaire
SUI: Stress urinary incontinence
TVT: Tension–free vaginal tape
UUI: Urge urinary incontinence
